# Optimizing synchronous extraction and antioxidant activity evaluation of polyphenols and polysaccharides from Ya'an Tibetan tea (*Camellia sinensis*)

**DOI:** 10.1002/fsn3.1331

**Published:** 2019-12-13

**Authors:** Qiaoran Zheng, Wenfeng Li, Heng Zhang, Xiaoxu Gao, Si Tan

**Affiliations:** ^1^ School of Advanced Agriculture and Bioengineering Yangtze Normal University Chongqing China; ^2^ Drug Control Institutions Ya'an Polytechnic College SiChuan China

**Keywords:** antioxidant activity, optimizing synchronous extraction, tea polyphenols, tea polyphenols compositions, tea polysaccharides, Ya'an Tibetan tea

## Abstract

The optimal synchronous conditions to extract tea polysaccharides (TPS) and tea polyphenols (TPP) from Ya'an Tibetan tea were investigated, and the antioxidative capacity of TPS and TPP was measured, and the tea was analyzed to identify the polyphenol compounds it contained. On the basis of single‐factor experiments, a Box–Behnken design and response surface methodology were applied to optimize the hot water extraction conditions. The optimal extraction technology was determined as extraction temperature of 83°C, time of 104 min, and liquid‐to‐material ratio of 41 ml/g, yielding TPP and TPS at 42.70 ± 2.38 mg/g and 53.86 ± 3.79 mg/g, respectively. The TPS and TPP in Ya'an Tibetan tea have high eliminating activities on DPPH and strong reducing power, with TPP showing a higher antioxidant activity than TPS. UHPLC‐QqQ‐MS/MS analysis identified EGCG, GCG, and ECG as major polyphenol components in Ya'an Tibetan tea. These findings might promote the application of Ya'an Tibetan tea in the food industry.

## Graphical abstract

Kangzhuan brick tea, also known as Tibetan tea, is a typical dark tea with a dark brown color. A highly efficient synchronous extraction process of tea polyphenols (TPP) and tea polysaccharides (TPS) in Tibetan tea was obtained in this study. Both TPS and TPP in Ya'an Tibetan tea have high antioxidant activities.

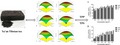

## 1 INTRODUCTION

Tea (*Camellia sinensis*) is one of the most popular beverages consumed worldwide. Tea is classified into green tea, oolong tea, black tea, and dark tea according to the fermentation level (Weerawatanakorn et al., 2015). Chinese dark tea is postfermented tea, the history of which dates back to the Ming Dynasty at 1,500 A.D. Tea was initially produced in Southwestern China and then carried via the mountains to Tibet and Xinjiang. In contrast to black tea, the process used for the fermentation of dark tea is known as “postfermentation” (or “wodui” in Chinese, meaning “pile‐fermentation” as a type of “controlled composting”), whereby these wet teas are compressed and formed into brick, mushroom, square, bowl, and cake shapes so as to be convenient for transportation. The bricks of tea then undergo controlled fermentation with a mixture of bacteria and mold that excludes disease‐causing microbes, and the polyphenols in tea also limit the growth of bacteria that can cause disease and spoilage while allowing the correct microbes to break down the material. With increasing fermentation, the color of the tea becomes darker, the tea softens in texture, and the flavor becomes concentrated and rich but is no longer astringent (Zhang, Zhang, Zhou, Ling, & Wan, 2013).

In China, there has been considerable interest in dark tea because of its fungal aroma and also its biological activity, including its antioxidation effects (Zheng, Wan, & Bao, 2015), anti‐obesity (Li et al., 2013; Oi, Hou, Fujita, & Yazawa, 2012), antihyperglycemic (Yamashita, Wang, Tinshun, Nakamura, & Ashida, 2012), and immunoregulation (Liu, Yu, Zhu, Zhang, & Chen, 2016). The various Chinese dark teas include Pu‐erh brick tea (Yunnan province), Fuzhuan brick tea (Hunan province), Kangzhuan brick tea (Sichuan province), Heimao tea (Hunan province), and Qingzhuan brick tea (Hubei Province) (Zheng et al., 2015).

Many studies have shown that tea polyphenols (TPP), theanine, and tea polysaccharides (TPS) are the main active substances in tea (Du et al., 2016). As the most biologically active group in tea, TPP possess a variety of therapeutic effects, such as antioxidant (Higdon & Frei, 2003; Mao, Gu, Chen, Yu, & He, 2017; Serafini, Ghiselli, & Ferro‐Luzzi, 1996), anti‐obesity (Uchiyama, Taniguchi, Saka, Yoshida, & Yajima, 2011; Xu, Hu, Hu, Wang, Wan, & Bao, 2015; Xu, Zhang, et al., 2015), antitumor (Afzal, Safer, & Menon, 2015), antihyperlipidemia (Huang, Zhang, et al., 2013; Huang, Chen, et al., 2013), and they also inhibit the activity of glucosidase and α‐amylase (Deng, Lin‐Shiau, Shyur, & Lin, 2015; Gao, Xu, Wang, Wang, & Hochstetter, 2013; Hara & Honda, 1990). TPP mainly include (−)‐epicatechin (EC), (+)‐gallocatechin (GC), (−)‐epigallocatechin (EGC), (−)‐epicatechin gallate (ECG), (−)‐epigallocatechin gallate (EGCG), and (+)‐gallocatechin gallate (GCG), and these phenols were oxidized and polymerized during microbial postfermentation period in Chinese dark tea (Luo et al., 2013).

Another important bioactive component in tea is TPS, although there has been a lack of studies on this substance. Recently, there have been studies showing that TPS has many health benefits including antioxidant, antitumor, antibacteria, anti inflammation, and detoxification (Chen et al., 2018; Li, Wang, Yuan, Pan, & Chen, 2018; Mao et al., 2018; Ren, Hu, Luo, & Yang, 2015).

Kangzhuan brick tea, also known as Tibetan tea, is a typical dark tea with a dark brown color. Tibetan tea is mainly produced in Ya'an, Sichuan, China and is the most popular drink for the Tibetan people. Tibetan tea has a history of thousands of years and contains multiple beneficial elements and bioactive substances. Tibetan tea is often consumed with high‐fat foods, such as beef and cheese, to promote better digestion of fat and cholesterol. However, there have been a few studies on the active ingredients of Tibetan tea, and to guide the effective use of Tibetan tea, further study is required.

There are many studies describing how to extract either TPP or TPS alone. However, to the best of our knowledge, combinative extraction of TPP together with TPS from Tibetan tea has not been reported, which limits its commercial development. TPP and TPS extractions are affected by many factors. In many different research fields, factor analysis and experimental design are used to determine effective parameters and improve performance (Bilgin, Elhussein, Özyürek, Güçlü, & Şahin, 2018). And response surface methodology (RSM) is often used to determine the optimal parameters in the extraction research.

The objective of present study was to optimize the conditions of simultaneous extraction of TPP and TPS in Tibetan tea and to evaluate the antioxidant activities of TPP and TPS and analyzed the TPP compounds using ultra‐high‐performance liquid chromatography–triple quadrupole‐mass spectrometry (UHPLC‐QqQ‐MS).

## 2 MATERIALS AND METHODS

### 2.1 Materials

Ya'an Tibetan tea was obtained from Sichuan ya'an zhougongshan tea industry co. LTD. Folin–Ciocalteu's phenol reagent was purchased from Shanghai Labaide Biotech Co., Ltd. DPPH and gallic acid was purchased from Sigma. The standards of studied phenolic compounds (protocatechuic acid, epicatechin, caffeic acid, epigallocatechin gallate, epicatechin gallate, gallic acid, fumalic acid, gallocatechin gallate, quercetin, kaempferol, rutin, and gallocatechin) were purchased from Shanghai Yuanye Bio‐Technology Co., Ltd. Ammonium formate and methanol are HPLC grade. Trichloroacetic acid and potassium ferricyanide were purchased from Solarbio Science & Technology Co., Ltd.

### 2.2 Extraction technological process and single‐factor experiment

**Figure 1:**
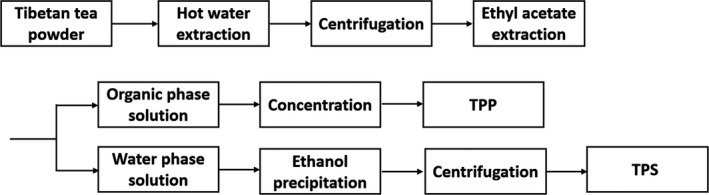
The synchronous extraction process of TPP and TPS

The synchronous extraction process that we employed to extract TPP and TPS is as Figure 1. Tibetan tea was ground until the particle size less than 355 μm. The single‐factor experiment for synchronous extraction was conducted, and the effect of extraction temperature (50, 60, 70, 80, 90°C), extraction time (60, 80, 100, 120, 140 min), and liquid/material ratio (20:1, 30:1, 40:1, 50:1, 60:1) on the extraction yield has been studied. For each tea extraction, distilled water was added to a flask containing 1 g of tea powder, and the extractions were performed in the given parameters. After the hot water extraction, the extracts were centrifuged at 3,000 *g*/min for 10 min. The supernatant was blended with the equal volume of ethyl acetate, and then, the organic phase was removed for determination of TPP. The aqueous phase was retained, to which 95% EtOH in a 1:4 (v/v) ratio at room temperature was added. The solution was left undisturbed for 12 hr and then collected the precipitate by 3,000 *g*/min centrifugation, and the precipitate was dried at 60°C to obtain TPS.

### 2.3 Response surface methodology (RSM)

Based on the results of the single‐factor experiments, a response surface methodology experiment was carried out to obtain the optimal synchronous extraction parameters, and the data were analyzed by Design‐Expert (8.0.6.1, Stat‐Ease Inc., USA). In the experiment, both extraction yield of TPS and TPP were used as the response value, and the liquid‐to‐material ratio (*X*_1_), extraction temperature (*X*_2_), and extraction time (*X*_3_) were independent variables (Table 2). The quadratic equation of the experiment is as follows:

$$Y=\beta_{0}+\sum_{i=1}^{3}\beta_{i}X_{i}+\sum_{i=1}^{3}\beta_{\mathrm{ii}}X_{i}^{2}+\sum_{i=1}^{2}\sum_{j=i+1}^{3}\beta_{\mathrm{ij}}X_{i}X_{j},$$

where *Y* denotes the response variables, *X_i_* and *X_j_* denote the independent variables, *β*_0_ is the intercept, *β_i_* is the linear, *β_ii_* is the quadratic, and *β_ij_* is the interaction coefficients.

### 2.4 Tea polyphenols content assays

The TPP content was analyzed by the Folin–Ciocalteu colorimetric method (Bilgin et al., 2018) with some modifications. First, 1.0 ml diluted sample was combined with 5.0 ml 10% Folin–Ciocalteu reagent. After 5 min, 4.0 ml of 7.5% Na_2_CO_3_ was added. After 1 hr, the TPP content was determined at 765 nm using a PT‐3502C microplate spectrophotometer (Potenov Co. Ltd.). The result was expressed as gallic acid equivalent per g of Tibetan tea (mg/g dry basis).

### 2.5 Tea polysaccharides content assays

The amount of TPS was determined using the phenol–sulfuric acid method (Guo, Guo, Zhu, & Jiang, 2016), and the result was expressed as glucose equivalent per g of Tibetan tea (mg/g dry basis).

### 2.6 UHPLC‐QqQ‐MS/MS analysis

Chromatographic separation coupled with mass spectrometry was performed with an ultra‐HPLC system (Agilent) and triple quadruple mass spectrometry (6460QqQ‐MS/MS; Agilent) equipped with an electrospray ionization source. For chromatographic elucidation, samples were injected onto a ZORBAX Eclipse Plus C18 column (50 mm × 2.1 mm i.d., 1.8 µm, Agilent) reversed phase packing column. The mobile phase consisted of 10 mmol/L ammonium formate (A) and methanol (B) at a flow rate of 0.4 ml/min, and the injection volume was 5 μl at 30°C column temperature. A gradient elution was this as follows: 90%–10% B in 6 min, 10% B in 7 min, 90% B in 7.5 min, and 90%–90% B in 10 min.

ESI in negative ionization mode was performed with the following parameters: the drying gas flow rate was 10.0 ml/min, gas temperature was set at 350°C, and capillary voltage was 3,500 V. A multiple reaction monitoring (MRM) mode was used, and all the transitions (Table 1) were monitored over the run time with dwell times of 20 ms.

**Table 1:** Identification of phenolic compounds found in TPP and tea soup using UHPLC‐QqQ‐MS/MS

Compounds	RT	MS [M−H]^−^	MS/MS (*m*/*z*)	CE (V)	Fragmentor (V)
Rutin	3.294	609	300	35	160
Quercetin	4.072	300.9	150.9, 120.8	20	140
Kaempferol	4.497	285	107, 160.9	40	140
Gallocatechin gallate	2.347	457	169, 304.9	10	140
Gallocatechin	1.083	305	125, 163	20	140
Gallic acid	0.361	169	124.9	10	120
Fumalic acid	1.248	193	133.7	15	100
Epigallocatechin gallate	2.085	457	169, 124.4	10	140
Epicatechin gallate	2.626	441	169, 289	15	140
Epicatechin	2.280	289	109, 124.6	25	140
Caffeic acid	0.710	179	134.9, 106.6	15	100
Protocatechuic acid	0.392	153	108.9	15	100

Abbreviations: CE, collision energy; RT, retention time (min); V, volt.

### 2.7 2,2‐Diphenyl‐1‐picrylhydrazyl (DPPH) radical scavenging assay

The DPPH assay was determined according to a previously published method with some modification (Guo et al., 2016). The samples were analyzed at 517 nm against a blank sample without DPPH. DPPH radical scavenging capacity was calculated as follows:

$$\text{Inhibition}\;\left(\%\right)=\left(1-\frac{A_{i}-A_{j}}{A_{c}}\right)\times 100$$

where *A_i_* is the sample with DPPH‐ethanol solution, *A_j_* is the absorbance of the sample with ethanol, and *A_c_* is the distilled water mix with DPPH‐ethanol solution.

### 2.8 Reducing power assay

The reducing power assay was determined following a reported method with slight modifications (Qiu, Wang, Song, Deng, & Zhao, 2018). The sample solution (100 μl) was mixed with 250 μl 0.2 mol/L phosphate buffer and 250 μl 1% potassium ferricyanide. The mixed solution was heated to 50°C for 20 min and then cooled rapidly. Then, 250 μl 10% TCA was added to the solution and centrifuged at 1580*g* for 10 min. Next, 250 μl of the supernatant was mixed with 250 μl distilled water and 50 μl 0.1% FeCl_3_, and the mixtures were measured at 700 nm using a PT‐3502C microplate spectrophotometer. The results were expressed as absorbance values.

### 2.9 Statistical analysis

All data are presented as the mean ± *SD* and subjected to LSD tests using the software SPSS 16.0 statistical software package (SPSS Inc., Chicago, IL, USA). *p* values < .05 were considered to be significant.

## 3 RESULTS AND DISCUSSION

### 3.1 Influence of extraction parameters on total tea polyphenol and tea polysaccharide yield

In the present study, the single‐factor experiment was adopted to study the effect of three extraction parameters (temperature, time, liquid‐to‐material ratio) on the extraction yield.

#### 3.1.1 Extraction temperature

**Figure 2:**
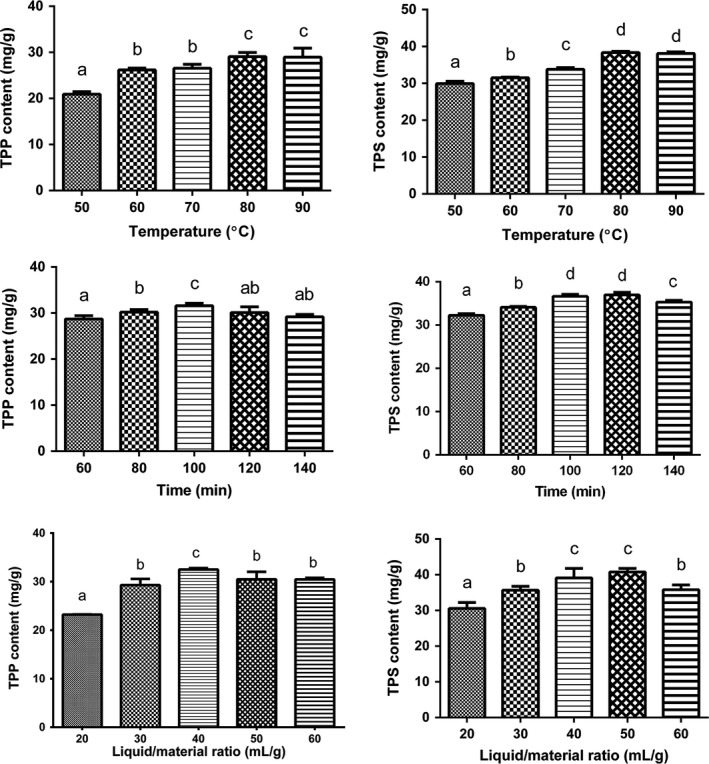
Influence of extraction temperature, extraction time, and liquid/material ratio on the extraction of TPP and TPS from Ya'an Tibetan tea, and columns not sharing a common letter showed significant difference (*p* < .05)

In the present study, the extraction yield of TPP and TPS was significantly affected by temperature (Figure 2). Moreover, extraction content of TPP reached the maximum when the temperature increased to 80°C from 50°C (*p* < .05); then, as the temperature continued to increase, there was no significant difference in TPP yield (*p* > .05). Similar results were found for Yunwu tea (Guo et al., 2016). The study indicated that high temperature benefited the dissolution and release of TPS and TPP in water by reducing the viscosity and improving the diffusion coefficient. The similar phenomenon was seen in TPS extraction. Therefore, 80°C was applied in RSM study.

#### 3.1.2 Extraction time

As shown in Figure 2, extraction time was significantly affected the yield of TPP and TPS. Saklar, Ertas, Ozdemir, and Karadeniz (2015) have also reported that the extraction time was the main factor when extracting TPP. In the early and middle stages of extraction, the yield of TPP and TPS increased as the duration of the extraction increased, and the maximum extraction yields of TPP and TPS were achieved at 100 min and 120 min, respectively. In the present study, the yield of TPP and TPS decreased at the later stage of extraction. For TPP, this may be explained by the exacerbated polyphenol oxidation as the extraction time increased (Pérez‐Burillo, Giménez, Rufián‐Henares, & Pastoriza, 2018). As for TPS, this may be due to the destruction of the structure of polysaccharides by the lengthy treatment at elevated temperature that resulted in a lower yield of polysaccharides (Guo et al., 2016). Thus, extraction time 100 min was chosen as the central point in RSM design.

#### 3.1.3 Liquid‐to‐material ratio

In the present study, extraction content of TPP in the 40:1 group was significantly higher than that in other groups (*p* < .05), and the ratio of 20:1 showed significantly lower yield than other groups (*p* < .05) (Figure 2). These results are in accordance with previous findings that a higher liquid‐to‐material ratio leads to a higher yield of polyphenols, and a higher liquid–material ratio generates a decrease in the consumption of tea material and decrease in the cost of extraction (Ćujić et al., 2016). However, when the ratio of liquid to material decreases to a certain extent, TPP are gradually saturated in the solution, and then the amount of dissolved TPP becomes low. As for TPS extraction, the extraction yield in the 40:1 group and 50:1 group was significantly higher than that in the other groups (*p* < .05). It is suggested that a high ratio of liquid to material accelerated the reaction between the surfaces of polysaccharides and the liquid, then resulted in the high yield of TPS (Cho, Getachew, Saravana, & Chun, 2019). Therefore, the liquid‐to‐material ratio of 40:1 was applied in the further study.

### 3.2 Fitting the model

In the present study, the effect of extraction temperature, extraction time and the ratio of liquid to material on the yield of TPP and TPS was investigated, and the corresponding results were shown in Table 2. The fitted second‐order Equations (1) and (2) are given as below:

(1) $$\text{TPP}=42.46+0.84X_{1}+5.43X_{2}+1.96X_{3}+2.07X_{1}X_{2}+1.77X_{1}X_{3}+1.64X_{2}X_{3}-7.40X_{1}^{2}-5.64X_{2}^{2}-5.94X_{3}^{2}$$

(2) $$\text{TPS}=53.52+3.21X_{1}+6.93X_{2}+2.18X_{3}-0.16X_{1}X_{2}+1.21X_{1}X_{3}-2.13X_{2}X_{3}-9.71X_{1}^{2}-14.16X_{2}^{2}-8.30X_{3}^{2}$$

**Table 2:** The actual levels of the operational parameters and observed values of Box–Behnken design

No.	*X*_1_	*X*_2_	*X*_3_	Response	
	L/M ratio (ml/g)	Temperature (°C)	Time (min)	TPP content (mg/g)	TPS content (mg/g)
1	30.00	70.00	100.00	25.97	21.11
2	50.00	70.00	100.00	23.03	25.16
3	30.00	90.00	100.00	31.66	34.46
4	50.00	90.00	100.00	37.00	37.87
5	30.00	80.00	80.00	28.21	29.05
6	50.00	80.00	80.00	26.83	35.75
7	30.00	80.00	120.00	27.86	32.84
8	50.00	80.00	120.00	33.55	44.40
9	40.00	70.00	80.00	24.24	20.34
10	40.00	90.00	80.00	32.86	39.28
11	40.00	70.00	120.00	25.62	27.11
12	40.00	90.00	120.00	40.79	37.52
13	40.00	80.00	100.00	42.34	53.52
14	40.00	80.00	100.00	43.90	52.81
15	40.00	80.00	100.00	41.14	54.22

The analysis of variance for the experimental results of the Box–Behnken design is presented in Table 3. The *p* value of TPP (*p* = .0002) and TPS (*p* = .0004) was significant, which suggested that the model was good fit with the experimental data. The *p* value for lack of fit was insignificant (*p* > .05). Moreover, the $R_{\text{Adj}}^{2}$ of TPP model and TPS model was 0.9895 and 0.9862, respectively, the results showed that the experimental data of RSM had a high degree of accuracy and reliability and that the observed and predicted data correlated well. Content of TPP was significantly affected by extraction temperature (*p* < .0001) and extraction time (*p* < .01), and the yield of TPP increased by increasing the extraction time. Furthermore, for the extraction of TPP, the liquid‐to‐material ratio had no remarkable effect on TPP yield (*p* < .05). The results also showed that all three extraction factors significantly affected the extraction rate of TPS (*p* < .05).

**Table 3:** ANOVA for the effect of temperature, time, and liquid‐to‐material ratio on the content of TPP and TPS using the response surface model

Source	*df*	TPP			TPS		
		Sum of squares	*F* value	*p* value	Sum of squares	*F* value	*p* value
Model	9	704.3978	52.18	.0002	1707.03	39.80	.0004
*X*_1_	1	5.628013	3.75	.1105	82.69	17.35	.0088
*X*_2_	1	235.9878	157.35	<.0001	383.78	80.53	.0003
*X*_3_	1	30.7328	20.49	.0062	38.06	7.99	.0368
*X*_1_ *X*_2_	1	17.1396	11.43	.0197	0.10	0.021	.8892
*X*_1_ *X*_3_	1	12.49623	8.33	.0343	5.90	1.24	.3163
*X*_2_ *X*_3_	1	10.72563	7.15	.0441	18.19	3.82	.1082
$X_{1}^{2}$	1	202.4641	134.99	<.0001	348.10	73.04	.0004
$X_{2}^{2}$	1	117.4508	78.31	.0003	740.02	155.29	<.0001
$X_{3}^{2}$	1	130.3876	86.94	.0002	254.18	53.34	.0008
Lack of fit	3	3.67	0.64	.6578	22.83	15.31	.0619

**Figure 3:**
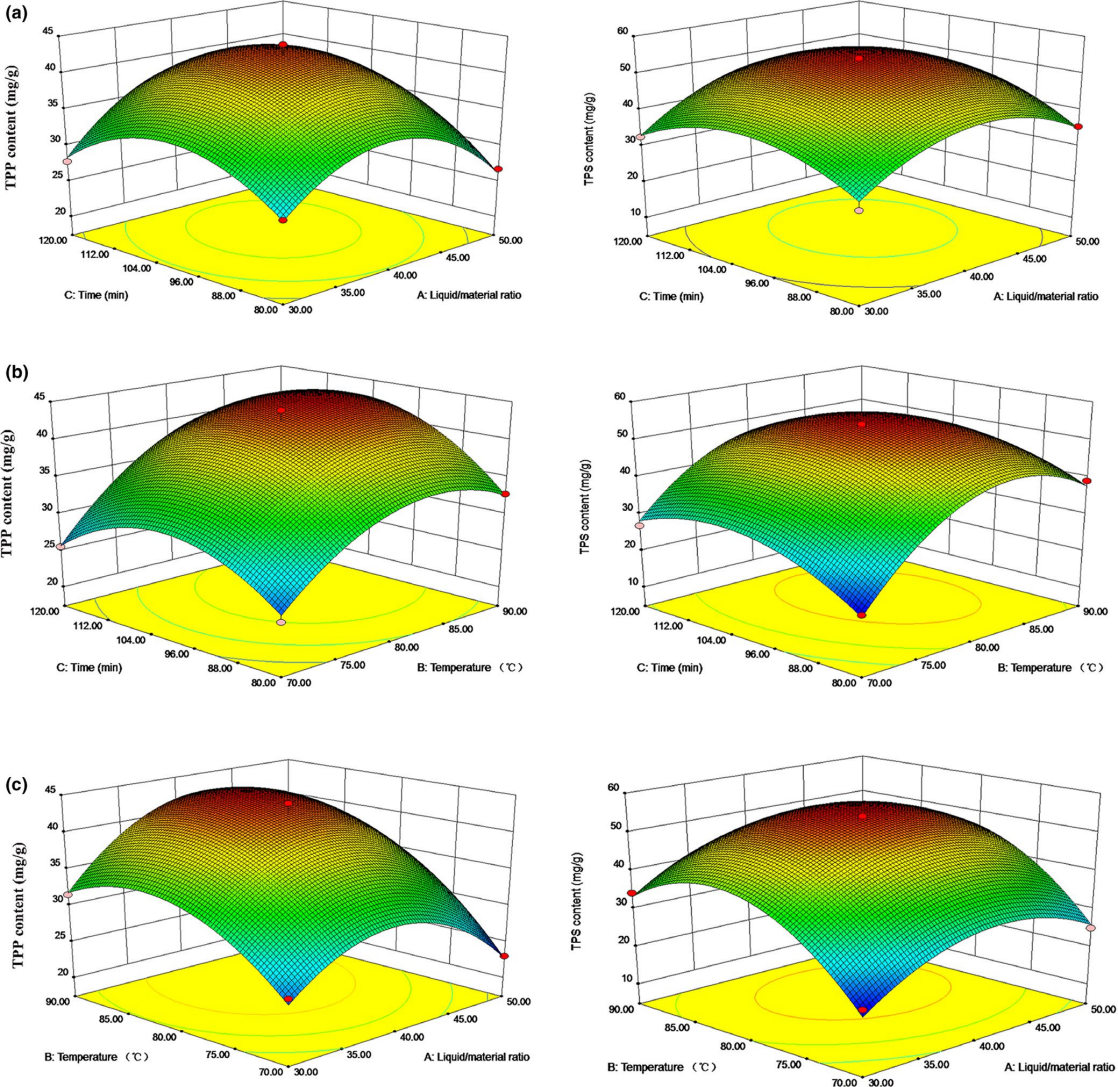
Response surface plots for the effects of temperature and time (a), temperature and liquid‐to‐material ratio (b), time and liquid‐to‐material ratio (c) on the yield of TPP and TPS

Response surface plots of TPP yield and TPS yield are shown in Figure 3a–c. The effect of extraction time and liquid‐to‐material ratio on the yield of TPP and TPS is shown in Figure 2a. The result showed that the interaction between extraction time and liquid‐to‐material ratio (*X*_1_ *X*_2_) showed a significantly positive effect on the TPP yield (*p* < .05); however, the TPS yield was not significantly affected by the interaction (*p* > .05). Moreover, interaction between *X*_1_ *X*_3_ and *X*_2_ *X*_3_ also had significant effect on the TPP extraction yield.

According to Equations (1) and (2), the optimized parameters were used for the synchronous extraction of TPP and TPS were liquid‐to‐material ratio (40.88:1 ml/g), extraction temperature (82.92°C), and extraction time (104.32 min). Under these conditions, we got the predicted maximum values for TPP content (43.92 mg/g) and TPS content (54.51 mg/g). To test the reliability and accuracy of the predicted models, a verification experiment was operated with the optimum parameters (liquid‐to‐material ratio 41:1 ml/g, extraction temperature 83°C, extraction time 104 min), as a result, the TPP and TPS content were, respectively, 42.70 ± 2.38 mg/g and 53.86 ± 3.79 mg/g in the verification experiments, and there was no significant difference between the predicted values and verification values (*p* > .05), which suggested that the fitted model for the response of TPP and TPS was suitable and reliable. Therefore, TPP and TPS from Ya'an Tibetan tea could be extracted efficiently under the optimized conditions.

### 3.3 Antioxidant activity of TPP and TPS from Tibetan tea

**Figure 4:**
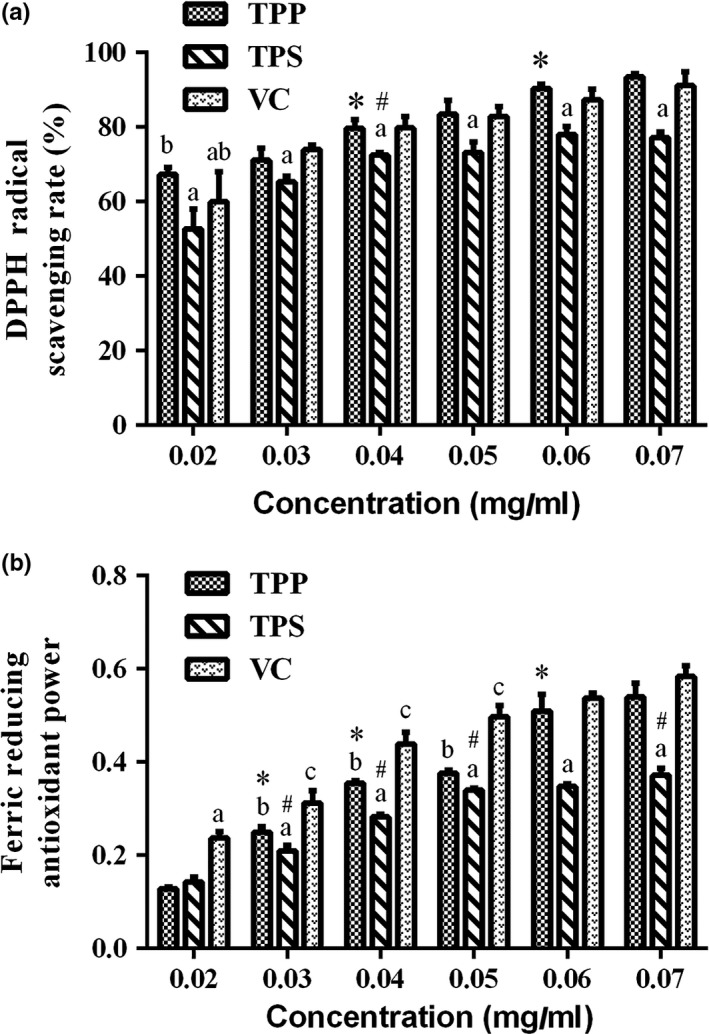
The antioxidant activity of Tibetan tea. A. DPPH radical scavenging activities of TPP, TPS, and Vc (mean ± *SD*, *n* = 3). Different letters (a, b, c) indicate significant differences (*p* < .05) between TPP, TPS, and Vc as the same concentration. Compared with the previous column data of TPP, an asterisk (*) represents *p* < .05. B. Ferric reducing antioxidant power of TPP, TPS, and Vc (mean ± *SD*, *n* = 3). Compared with the previous column data of TPS, an asterisk (#) represents *p* < .05

In the present study, the DPPH assay and reduction power assay were carried out to compare the antioxidant activity of TPP, TPS, and Vc in the range of 0.02–0.07 mg/ml. Both DPPH assay and reduction power assay are widely used to evaluate the antioxidant activity in active ingredients for food (Guo et al., 2014). As a very stable radical, DPPH appears purple when dissolved in ethanol and has a maximum absorption peak at 517 nm, and the stronger the scavenging ability of antioxidant, the lower the absorbance (Wang, Chen, Jia, Tang, & Ma, 2012; Wang, Yang, & Wei, 2012). Figure 4a shows that TPP possesses stronger scavenging of DPPH, and the effects depend on the dose in the range of experimental concentrations (*p* < .05). Moreover, there was no significant difference between TPP and Vc (*p* > .05), and the DPPH scavenging activity of TPS was lower than that of TPP and Vc for the concentrations of 0.02–0.07 mg/ml (*p* < .05). Other dark tea, such as Fuzhuan tea, Pu‐erh tea and Liubao tea, also displayed high DPPH radical scavenging ability (Lv, Zhang, Shi, & Lin, 2017).

The antioxidant activity also could be evaluated by reducing power in several research. In the assay, the Fe^3+^ of ferricyanide complex could be reduced to the Fe^2+^ by the antioxidant, and then, the potassium ferrocyanide is further reacted with FeCl_3_ to form Perl's Prussian blue with the maximum absorption peak at 700 nm (Guo et al., 2016). As shown in Figure 4b, the reducing power of TPP and TPS increased with concentration, and the activity of TPP was significantly stronger than that of TPS in the tested concentrations of our experiment (*p* < .05). Furthermore, the reducing power of TPP and TPS was lower than that of Vc for all of the concentrations tested in the present study.

Many studies have confirmed the strong antioxidant activity of TPP. High antioxidant activity has been observed for extracts of Pu‐erh tea (Huang, Zhang, et al., 2013; Huang, Chen, et al., 2013). Fan et al. (2013) also reported that Zijuan Pu‐erh tea reduced lipid‐membrane oxidation by scavenging free radicals. Moreover, Li, Chen, Zhu, and Meng (2017) investigated the antioxidant activity of TPP from three dark tea and found that Ya'an Tibetan tea showed the highest antioxidant activity. TPS from brick tea also exhibited dose‐dependent antioxidant activity (Yang et al., 2017), and it was reported that the DPPH radical scavenging activities of TPS could be attributed to the carboxyl group in hexuronic acid (Wang, Chen, et al., 2012; Wang, Yang, et al., 2012). Due to the difference of the tea processing technology and origin, there were also found significant differences in the composition of TPS, and affecting its antioxidant activity. In the fermentation process, antioxidant activity of deeply fermented oolong tea increased by the conjugation between its polysaccharide and protein (Wang, Chen, et al., 2012; Wang, Yang, et al., 2012). However, Zhao, Huangfu, Dong, & Liu, (2014) reported that TPS extracted from nonfermented green tea had stronger antioxidant activity than that extracted from fully fermented black tea. The changes in the antioxidant activity of TPS in Tibetan tea during fermentation require further study.

### 3.4 UHPLC‐QqQ‐MS analysis

**Figure 5:**
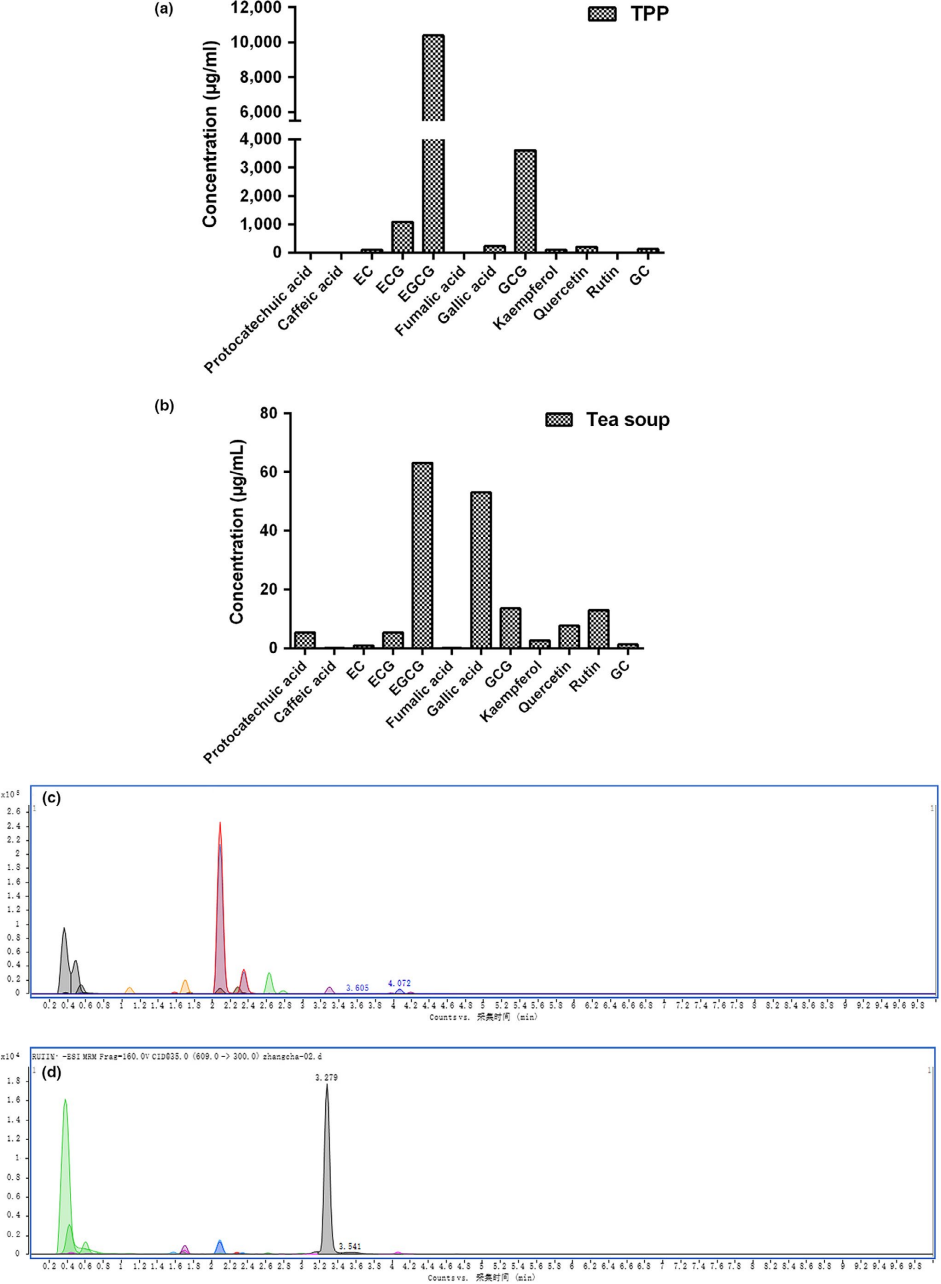
Concentrations of 12 compounds in TPP (a) and tea soup (b) and Chromatogram of 12 compounds in TPP (c) and tea soup (d)

Green tea exhibits strong bioactivity mainly because of its abundance of TPP, such as EGCG (Hamilton‐Miller, 1995). It was pointed out the total amount of polyphenols in tea is inversely proportional to the degree of fermentation. Along with the fermentation, the constituents and content of TPP were altered significantly by oxidation and polymerization in dark tea. We determined that there were 12 compounds in both TPP and tea soup of Ya'an Tibetan tea, including protocatechuic acid, epicatechin (EC), epigallocatechin gallate (EGCG), epicatechin gallate (ECG), fumalic acid, gallic acid (GA), gallocatechin gallate (GCG), gallocatechin (GC), caffeic acid, kaempferol, quercetin, and rutin (Figure 5a–d). Consistent with other studies, EGCG, GCG, ECG, and GA were identified as the major components in both TPP and tea soup of Ya'an Tibetan tea. In Fuzhuan brick tea, the content of EGCG, EGC, and ECG was much higher as compared to other catechins (Zhu et al., 2015). EGCG and ECG were also noted to be the predominant active compounds in brick tea (Zheng et al., 2015). Furthermore, as the major components of TPP, EGCG, GCG, and ECG were mainly responsible for the antioxidant activity of tea (Guo et al., 2016). In tea, the significant positive correlation between the content of EGCG and the antioxidant activity has also been proven by the FRAP and ABTS experiments (Lv et al., 2017; Xu, Hu, et al., 2015; Xu, Zhang, et al., 2015). These studies indicate that the antioxidant activity of Tibetan tea might be attributed to its polyphenolic components, such as EGCG, GCG, and ECG.

With the development of the fungal postfermentation, the content of galloyl catechins decreased gradually, especially EGCG and ECG (Zhu et al., 2015). The reduction of catechin in microbial fermented tea mainly due to polymerization and oxidative cleavage of aromatic rings, and then, the humic acids were produced (Zheng et al., 2015). Similarly, the level of EGCG was significantly higher in raw Pu‐erh tea than that of fermented Pu‐erh tea (Chen et al., 2018).

Interestingly, in the present study, the level of EGCG in Ya'an Tibetan tea was significantly higher than that in other brick teas, and it was suggested that the EGCG in Ya'an Tibetan tea was poorly degraded by microorganisms during the fermentation process when the raw dark tea turned into dark tea. For the postfermented tea, microorganisms play a key role in tea quality. Chen et al. (2018) found *Aspergillus cristatellus* present in Fuzhuan brick tea produced in Shanxi province. In Pu‐erh tea, the main microbes were *Rhizopus*, *Saccharomyces*, *Aspergillus*, *Penicillium*, and *Bacterium* (Zhao et al., 2013). Therefore, we consider that during the fermentation stage, the main microbes in Ya'an Tibetan tea may be different from those in other brick tea. However, because the microbiological method of action is unknown during the pile‐fermentation processing, the specific mechanism requires further study.

Moreover, the concentrations of these polyphenols in TPP were considerably higher than that in tea soup. It was suggested that TPP can be obtained more effectively through the RSM extraction process. Therefore, effective extraction technology is useful for the most efficient extraction of TPP from Ya'an Tibetan tea.

In addition, EGCG, GCG, and ECG in tea also have many biological properties, such as immune activity, antimutagenic, antidiabetic, and antibacterial activity (Puligundla, Mok, Ko, Liang, & Recharla, 2017), and EGCG is directly correlated with several positive effects on human health, including anti‐inflammatory, anticancer, and anti‐atherosclerotic activity (Tenore, Daglia, Ciampaglia, & Novellino, 2015); therefore, other activities of Ya'an Tibetan tea should be further developed.

## 4 CONCLUSION

A Box–Behnken design based on RSM was demonstrated to be sufficient to optimize conditions for the hot water extraction of TPP and TPS from Ya'an Tibetan tea, and the liquid‐to‐material ratio, temperature, and time had significant effects on the extraction. The highest yields of TPP and TPS were obtained with a liquid‐to‐material ratio of 41 ml/g, extraction temperature of 83°C, and extraction time of 104 min. Furthermore, the DPPH assay and reduction power assay indicated that both TPP and TPS of Tibetan tea possessed strong antioxidant capability, with TPP showing higher antioxidant activity than TPS. Moreover, EGCG, GCG, ECG, and GA were identified as major polyphenol components in both TPP and tea soup from Ya'an Tibetan tea. These findings are beneficial to the development and application of Ya'an Tibetan tea in the food industry.

## CONFLICT OF INTEREST

The authors declare that there is no conflict of interests.

## ETHICAL APPROVAL

This study does not involve any human or animal testing.

## INFORMED CONSENT

Written informed consent was obtained from all study participants.
